# The Base Excision Repair System of *Salmonella enterica* serovar Typhimurium Counteracts DNA Damage by Host Nitric Oxide

**DOI:** 10.1371/journal.ppat.1000451

**Published:** 2009-05-29

**Authors:** Anthony R. Richardson, Khanh C. Soliven, Margaret E. Castor, Penelope D. Barnes, Stephen J. Libby, Ferric C. Fang

**Affiliations:** 1 Department of Laboratory Medicine, University of Washington, Seattle, Washington, United States of America; 2 Department of Microbiology, University of Washington, Seattle, Washington, United States of America; 3 Department of Medicine, University of Washington, Seattle, Washington, United States of America; The Rockefeller University, United States of America

## Abstract

Intracellular pathogens must withstand nitric oxide (NO·) generated by host phagocytes. *Salmonella enterica* serovar Typhimurium interferes with intracellular trafficking of inducible nitric oxide synthase (iNOS) and possesses multiple systems to detoxify NO·. Consequently, the level of NO· stress encountered by *S.* Typhimurium during infection *in vivo* has been unknown. The Base Excision Repair (BER) system recognizes and repairs damaged DNA bases including cytosine and guanine residues modified by reactive nitrogen species. Apurinic/apyrimidinic (AP) sites generated by BER glycosylases require subsequent processing by AP endonucleases. *S*. Typhimurium *xth nfo* mutants lacking AP endonuclease activity exhibit increased NO· sensitivity resulting from chromosomal fragmentation at unprocessed AP sites. BER mutant strains were thus used to probe the nature and extent of nitrosative damage sustained by intracellular bacteria during infection. Here we show that an *xth nfo S.* Typhimurium mutant is attenuated for virulence in C3H/HeN mice, and virulence can be completely restored by the iNOS inhibitor L-NIL. Inactivation of the *ung* or *fpg* glycosylase genes partially restores virulence to *xth nfo* mutant *S.* Typhimurium, demonstrating that NO· fluxes *in vivo* are sufficient to modify cytosine and guanine bases, respectively. Mutants lacking *ung* or *fpg* exhibit NO·–dependent hypermutability during infection, underscoring the importance of BER in protecting *Salmonella* from the genotoxic effects of host NO·. These observations demonstrate that host-derived NO· damages *Salmonella* DNA *in vivo*, and the BER system is required to maintain bacterial genomic integrity.

## Author Summary

The host innate immune system represents the first line of defense against invading microorganisms. An integral part of this response involves the production of nitric oxide (NO·), a potent antimicrobial effector. Bacterial pathogens have evolved a variety of systems to counteract the inhibitory effects of host NO·. Here we describe the role of a complex DNA repair pathway in the bacterium *Salmonella* Typhimurium that minimizes the mutagenic nature of host NO·. We have determined the mutation rate of bacteria during a model infection. Our results reveal that the *Salmonella* Base Excision Repair system (BER), comprised of DNA glycosylases and AP endonucleases, is able to eliminate excess mutations that accumulate in NO·–exposed cells during their interaction with the host. Thus, during *Salmonella* infections, the BER system protects the bacterium against potentially detrimental DNA damage arising from NO·–exposure. This provides genomic stability to a pathogenic microorganism that has evolved to survive within the genotoxic intracellular environment of host phagocytes.

## Introduction

Host innate immunity represents the first line of defense against invading pathogenic microorganisms. Nitric oxide (NO·) is an essential component of this innate immune system, which is required for the efficient clearance of pathogenic fungi, viruses, parasites and bacteria [1],[2]. Inflammatory NO· is produced by the inducible Nitric Oxide Synthase (iNOS) of activated phagocytes [3]. NO· exposure can inhibit bacterial growth through the modification of multiple intracellular targets including protein thiols, heme containing proteins, thiol-coordinated metals, lipid bilayers, and DNA [4]–[8]. The Type III Secretory System (TTSS) of *S.* Typhimurium encoded on *Salmonella* Pathogenicity Island 2 (SPI2) impedes trafficking of iNOS to the *Salmonella* Containing Vacuole (SCV) in host macrophages [9]. In addition, NO· is detoxified by the Hmp flavohemoglobin, which is required for virulence in hosts proficient for inflammatory NO· production [10]. Thus, *Salmonella* has evolved multiple mechanisms to limit bacterial NO· exposure during infection, and consequently the degree of nitrosative stress to which *S.* Typhimurium is subjected *in vivo* is unknown.

Although NO· does not directly damage DNA, NO· congeners such as nitrous anhydride (dinitrogen trioxide, N_2_O_3_) or peroxynitrite (ONOO^−^) are capable of directly modifying nucleic acids [8],[11]. Nitrous anhydride generated from the spontaneous NO· autooxidation is a potent deaminating species of the DNA bases guanine, adenine, and cytosine, to produce xanthine (dX), hypoxanthine (dHX), and uracil (dU), respectively [8]. Unless repaired, dU, dX and dHX in a DNA molecule are highly mutagenic resulting in transition mutations, i.e., GC→AT or AT→GC. Peroxynitrite produced by the reaction of NO· and superoxide (O_2_^−^) is an oxidant that can preferentially target guanine residues in DNA to produce mutagenic 8-oxoguanine and unstable 8-nitroguanine residues [11],[12]. Moreover, the increased reactivity of 8-oxoguanine towards peroxynitrite can produced secondary cytotoxic oxidation products [11]. Thus, cells exposed to high concentrations of host NO· must respond to both mutagenic and cytotoxic DNA lesions. However, whether host-derived NO· is capable of promoting mutagenesis of intracellular *S.* Typhimurium has not been determined.

The base excision repair (BER) pathway has proven to play a critical role in the defense against the deleterious effects of NO·. BER involves the recognition of modified bases by specific DNA glycosylases, which cleave the N-glycosidic bonds of damaged bases to release them from the phosphodiester DNA backbone. In enteric bacteria, several DNA glycosylases are responsible for the removal of damaged DNA bases. Following base deamination, Uracil DNA Glycosylase (Ung) is required for the removal of dU, and 3-methyladenine DNA glycosylase (AlkA) can remove dX and dHX residues [13],[14]. Oxidized guanines (i.e., 7,8-dihydro-8-oxodeoxyguanine (8-oxoG) and formamidopyrimidine (FapyG)) are processed by Formamidopyrimidine DNA glycosylase (Fpg), which can also recognize hypoxanthine and xanthine, albeit with lower affinity [15],[16]. A related enzyme, Endonuclease VIII (Nei), removes oxidized pyrimidines, although Nei can also exhibit activity towards FapyG and 8-oxoG [17]. Finally, Endonuclease III (Nth) repairs oxidized and ring-saturated pyrimidine bases, although these lesions are not typically associated with nitrosative stress [18]. The AP sites resulting from glycosylase-mediated base removal cannot be acted upon directly by DNA polymerase and may consist of altered DNA ends such as a 3′-PO_4_ or a 3′-phospho-α,β-unsaturated aldehyde. Rather, glycosylase-generated AP sites must first be processed by one of two AP endonucleases in enteric bacteria, endonuclease IV (Nfo) and exonuclease III (Xth), the latter of which comprises ∼90% of total cellular AP endonuclease activity [19],[20]. AP endonuclease processing leaves 5′-PO_4_ and 3′-OH DNA ends that can be repaired by DNA polymerase I (PolA) and ligase.

Cells devoid of AP endonuclease activity (*xth nfo*) have been shown to be hypersusceptible to ionizing radiation, oxidative stress and NO· exposure in *Escherichia coli* and *Salmonella enterica* serovar Typhimurium [21],[22]. Without AP endonuclease activity, glycosylase-generated AP sites can persist and yield more serious single- and double-strand breaks, lesions known to be toxic to rapidly dividing cells [23],[24]. It has been hypothesized that glycosylases recognize and remove NO·-modified bases, resulting in an accumulation of AP sites that reduces the viability of nitrosatively-stressed *xth nfo* mutant bacteria [23]. Indeed, inactivation of specific glycosylases in *E. coli*, particularly *ung* and *fpg*, partially reverses the NO· sensitivity of *xth nfo* mutants suggesting that elimination of the source of excess AP sites is beneficial to nitrosatively stressed cells devoid of AP endonuclease activity [23]. Moreover, NO· treatment of *E. coli* results in increased RecA-dependent recombination that can be ameliorated by further inactivation of *ung* or *fpg* [23]. Thus, NO·-stressed cells appear to suffer an abundance of double-strand breaks that presumably arise from unprocessed AP sites.

Previous studies of wild type *S.* Typhimurium failed to provide evidence of direct DNA damage in the intramacrophage environment [25]. However, Suvaranapunya and Stein found that *xth nfo* mutant *S.* Typhimurium exhibits impaired survival in cultured macrophages and a competitive defect in mice, implying that base damage occurs during *Salmonella*-host cell interactions [22]. The macrophage survival defect was dependent on the production of reactive oxygen or nitrogen species, as it was not observed in macrophages deficient in both the NADPH phagocyte oxidase and inducible NO· synthase (*phox* ^−/−^ *iNOS* ^−/−^ C57BL/6 peritoneal elicited macrophages) [22]. Thus, host innate immunity appears to cause DNA base damage in *S.* Typhimurium in the intracellular environment. However, these investigators failed to establish a specific contribution of NO·, nor did they determine whether excess AP sites in *xth nfo* mutant *S.* Typhimurium arise spontaneously or result from glycosylase-mediated repair of damaged bases during infection. Given that many DNA glycosylases (*e.g.* Fpg, Nei and Nth) can be inactivated by NO·-exposure, the ability of these enzymes to contribute to the generation of AP sites in the presence of NO· has been questioned [26]–[28].

The present study extends earlier work in the context of the host-pathogen interaction by exploiting the NO·-sensitivity and attenuated virulence of *xth nfo* mutant *S.* Typhimurium to probe the mechanism of DNA base damage during infection. In addition, the effects of NO· on bacterial mutation rates within the host environment have been determined. Our observations demonstrate for the first time that host-derived reactive nitrogen species are able to cause DNA base damage in intracellular bacteria, leading to the glycosylase-mediated generation of AP sites. More importantly, while BER is dispensable for *S.* Typhimurium to cause acute lethal infection in mice, this pathway is sufficient to limit all mutagenic effects associated with host-generated NO·.

## Results

### NO· exposure results in DNA base damage that is targeted by the Base Excision Repair (BER) system

**Figure 1:**
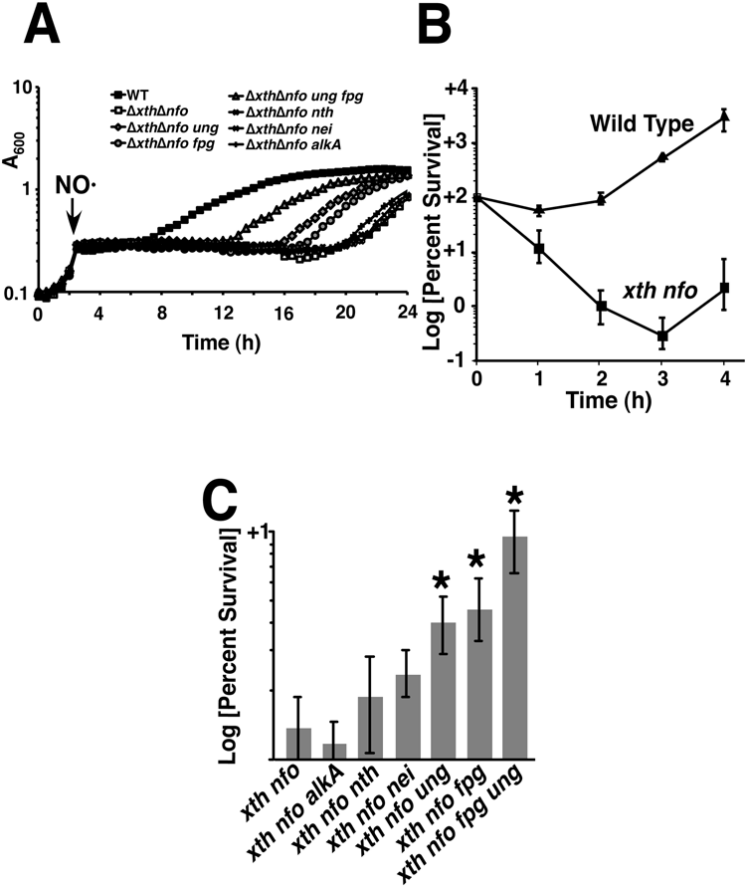
NO·–sensitivity of *S.* Typhimurium Base Excision Repair mutants. (A) Growth of *S.* Typhimurium 14028s and mutant derivatives after the addition of NO· from 2 mM SperNO (at arrow). (B) Survival of Wild Type and *xth nfo* after exposure to 1 mM SperNO. (C) Rescue of *xth nfo* cells by additional inactivation of indicated glycosylases after 2 hr exposure to 1 mM SperNO. Asterisks represent statistical significance. (p<0.05.)

An *S.* Typhimurium *xth nfo* mutant lacking AP endonuclease activity is hypersensitive to NO·, as demonstrated by a ∼12 h growth lag following the addition of 1 mM Spermine NONOate (SperNO) (Figure 1A). The extended lag period is attributable to increased cell death observed in NO·-exposed *xth nfo* mutants as compared to wild type cells (Figure 1B). The *xth nfo* mutant was killed rapidly following administration of SperNO, and cell killing continued for three hours until the NO· had been detoxified (Figure 1B). This directly contrasts with the bacteriostatic actions of NO· on wild type cells (Figure 1B). Additional inactivation of the *ung* or *fpg* glycosylase genes in *xth nfo S.* Typhimurium reduced the extended lag interval by 5 h and 4 h, respectively, and significantly enhanced survival during nitrosative stress (Figure 1A and 1C). Moreover, the ameliorative effects of *ung* or *fpg* inactivation on the NO·-sensitivity of the *xth nfo* mutant were additive, as demonstrated in an *ung fpg xth nfo* mutant strain (Figure 1A and 1C). In contrast, inactivation of *nei* or *nth* had little effect on the NO·-sensitivity of *xth nfo* mutants, suggesting that pyrimidine oxidation does not occur readily in NO·-exposed DNA (Figure 1A and 1C). Inactivation of *alkA* did not improve the survival of *xth nfo* cells during nitrosative stress, consistent with previous observations in *E. coli* [23], even though xanthine and hypoxanthine residues are known to accumulate in DNA following NO·-exposure [29]. Collectively, these data indicate that growth arrest correlates with decreased cell survival following NO·-exposure (Figure 1A and 1C). The enhanced NO·-susceptibility of *xth nfo S.* Typhimurium appears to be a consequence of the accumulation of lethal Ung- and Fpg-generated AP sites during the repair of NO·-exposed DNA.

### Attenuated virulence of *xth nfo* mutant *S.* Typhimurium results from NO·–mediated DNA base damage during infection

**Figure 2:**
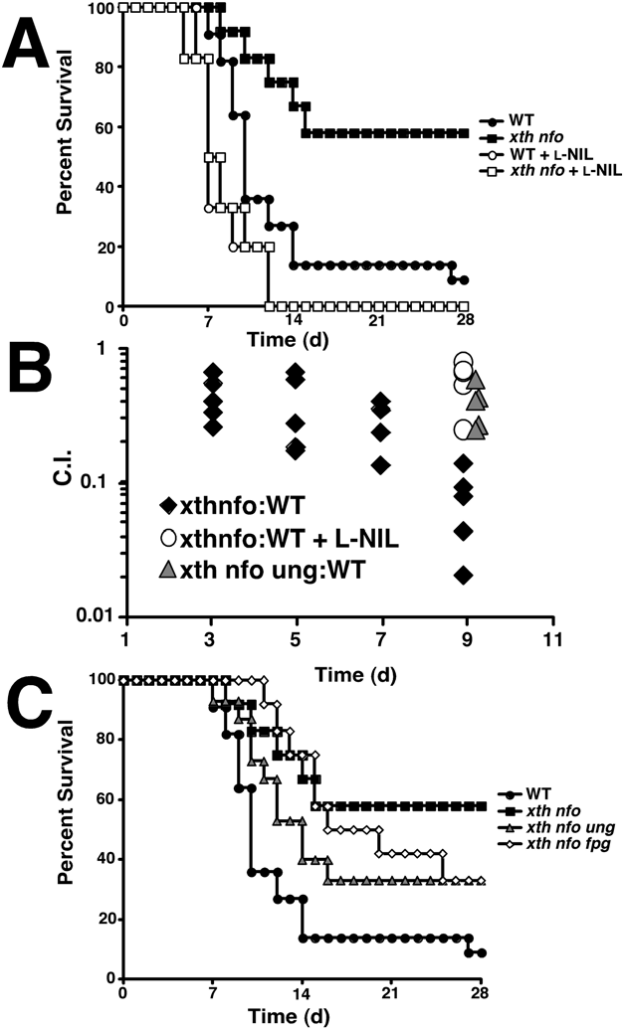
The attenuation of *xth nfo* mutant *S.* Typhimurium is due to NO·–mediated base damage. (A) Full virulence can be restored to *xth nfo S.* Typhimurium (14028s) in C3H/HeN mice by administration of the NOS2 inhibitor L-NIL. (B) Livers were harvested from female C3H/HeN infected with a mixed inoculum of Wild Type and mutant *S.* Typhimurium at indicated days. Ratios of mutant to Wild Type viable cfu·g^−1^ were determined and C.I. = Mutant_OUT_:WT_OUT_/Mutant_IN_:WT_IN_. (C) The attenuation of *xth nfo S.* Typhimurium is suppressed by further inactivation of DNA glycosylases *ung* and *fpg*.

C3H/HeN mice infected i.p. with 10^3^ cfu of *S.* Typhimurium 14028s or isogenic mutant derivatives demonstrated the attenuated virulence of an *xth nfo* mutant (Figure 2A and 2B). Virulence was completely restored to the *xth nfo* strain by oral administration of the NOS2-specific inhibitor L-NIL [L-N^6^-(1-iminoethyl)-lysine] [30] (Figure 2A). Furthermore, the competitive defect of *xth nfo* mutant versus wild type cells was abrogated by the inhibition of NO· production (Figure 2B). In concordance with the *in vitro* observations, the inactivation of *ung* or *fpg* increased the virulence of an *xth nfo* mutant, indicating that host-derived NO· leads to enzymatically-generated chromosomal AP sites (Figure 2B and 2C). The additional inactivation of other DNA glycosylases such as *nei* and *nth* had no effect on the virulence of an *xth nfo* mutant (data not shown). Although the inactivation of *ung* significantly increased the virulence of an *xth nfo* mutant strain, the *ung* mutation failed to restore full wild type virulence. This is best rationalized by the attenuating effects of an *ung* mutation in isolation; *ung* and *xth nfo ung* mutants display comparable virulence phenotypes (data not shown). The explanation for the effects of an isolated *ung* mutation on *Salmonella* virulence is presently unknown. Nevertheless, it can be concluded that Fpg and Ung together are responsible for a significant fraction of NO·-induced chromosomal AP sites during *S.* Typhimurium infection of murine hosts, making AP endonucleases (Xth and Nfo) essential in this setting.

### Ung and Fpg protect cells from the mutagenic effects of NO· exposure

Treatment of wild type *S.* Typhimurium with authentic NO· (from the NO· donor SPER/NO) resulted in a ∼14-fold increase in the rate of resistance to rifampin (Table 1). Exposure to peroxynitrite (ONOO^−^) or other oxidants generated by SIN-1 (3-morpholinosydnonimine) increased rifampin resistance rates by two-fold. SIN-1 can promote metal-catalyzed oxidative damage in addition to peroxynitrite-mediated cytotoxicity [31]. However, no effect of iron chelation was observed when this experiment was repeated in the presence of SIN-1 and 2,2′-dipyridyl (data not shown). *S.* Typhimurium *ung* mutants exhibited hypermutability even in the absence of NO·, reflecting basal levels of spontaneous cytosine deamination. However, following NO· exposure, *ung* mutants displayed mutation rates more than 50% higher than wild type cells (Table 1). In contrast, SIN-1 treatment did not significantly affect the mutability of *ung* cells, whereas SIN-1 doubled the mutation rates in wild type *S.* Typhimurium and quadrupled mutation rates in *fpg* mutant cells. These observations are consistent with differential targeting by various reactive nitrogen species. NO·-mediated deamination of cytosine *in vitro* results in C→T transitions that are counteracted by Ung. Similarly, peroxynitrite-dependent guanine oxidation results in G→T transversions that appear to be limited by Fpg.

**Table 1:** Mutability of *S.* Typhimurium strain 14028s derivatives upon exposure to nitric oxide (NO·) or peroxynitrite (ONOO^−^).

	Untreated	NO·	ONOO^−^
Wild Type	2.35	32.3^a^	4.45
*ung*	9.51	48.1a,b	8.64
*fpg*	2.34	32.0^a^	9.58a,b

Frequency of Rif^R^×10^−8^.

a Mutability significantly higher than unexposed cells (Wilcoxon Rank Sum, p≤.05).

b Mutability significantly higher than exposed WT cells (Wilcoxon Rank Sum, p≤.05).

**Figure 3:**
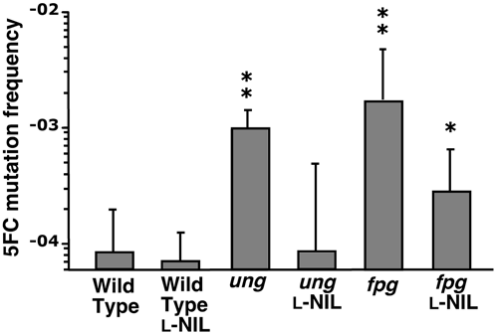
The base excision repair system of *S.* Typhimurium protects against the mutagenic effects of host NO· during infection. Bacteria isolated from livers 7d post inoculation were diluted and plated to determine total viable cfu·g^−1^, and 5FC resistance rates were determined as described in Materials and Methods. For comparison, C3H/HeN mice were administered L-NIL orally to inhibit NO· production. Asterisks indicate rates significantly different compare to Wild Type cells from untreated mice as determined by Wilcoxon Rank Sum test.( * p = 0.05 **p = 0.01.).

Rates of spontaneous resistance to conventional antibiotics like rifampin are too low (∼5×10^−8^) to be readily used as a measure of mutation frequency *in vivo* since total pre-terminal bacterial burdens in the murine *Salmonella* model only modestly exceed 10^6^ cfu/organ. An assay based on resistance to the antifungal agent 5-fluorocytosine (5-FC) was therefore used to measure bacterial mutation rates *in vivo*. 5-FC resistance results from loss-of-function mutations in the *codBA* genes that encode a cytosine permease and deaminase, respectively [32],[33]. Mutations that inactivate *codA* or *codB* prevent transport of the base analog into the cell or conversion to the highly toxic metabolite 5-fluorouracil, respectively. Measurement of 5-FC resistance in wild type *S.* Typhimurium recovered from the livers of infected mice demonstrated that host NO· does not exert mutagenic effects on *Salmonella* during infection; i.e., inhibition of host NO· production did not reduce observed 5-FC resistance rates (Figure 3). In contrast to wild type cells, NO·-mediated hypermutability was observed in *S.* Typhimurium strains lacking Ung or Fpg. In the case of *ung* mutant cells, the 12-fold excess in 5-FC mutation rate observed *in vivo* was entirely attributable to host NO·, as the administration of L-NIL completely eliminated hypermutability (Figure 3). In contrast, the 21-fold increase in 5-FC resistance exhibited by *fpg* mutant cells was only partially a consequence of DNA damage by host NO· (Figure 3). Treatment of *fpg* mutant-infected mice with L-NIL reduced bacterial mutability by 6-fold, although this mutant strain still exhibited 5-FC resistance rates significantly higher than those of wild type cells. This suggests that Fpg also plays a role in limiting mutations caused by NO·-independent mediators in the host environment, e.g., reactive oxygen species produced by the NADPH phagocyte oxidase.

## Discussion

A hallmark of *Salmonella* pathogenesis is the organism's ability to survive and replicate within the vacuoles of professional phagocytic cells. In this environment, *Salmonella* is exposed to cytotoxic NO· (or its congeners) produced by inducible NO· synthase (iNOS, NOS2). Thus, *S.* Typhimurium harbors many factors that allow it resist the cytotoxic effects of NO· and its oxidized congeners (e.g., the Hmp flavohemoglobin) [10]. This work demonstrates that *Salmonella* is additionally capable of avoiding the mutagenic effects of host NO· and maintaining genomic stability despite the highly genotoxic host environment. Mutant strains lacking specific elements of the Base Excision Repair (BER) pathway, in combination with the measurement of *in vivo* mutation rates, have allowed us to demonstrate NO·-dependent bacterial DNA damage in a murine infection model.

*S.* Typhimurium *xth nfo* mutants lacking AP endonuclease activity exhibit hypersusceptibility to NO· as well as to a variety of DNA damaging treatments including reactive oxygen species, alkylating agents, and UV- or Γ-irradiation. Each of these conditions generates an excess of chromosomal AP sites resulting either from direct modification of nucleic acids or the actions of various BER glycosylases. The accumulation of AP sites in an *xfo nth* mutant ultimately results in double strand breaks and chromosomal fragmentation. The NOS-dependent attenuation of *S*. Typhimurium *xth nfo* mutants during infection indicates that exposure to host-derived NO· is sufficient to damage bacterial DNA and generate AP sites (Figure 2). Previous work suggested that *Salmonella* DNA damage during infection might be attributable to either NO· or O_2_^−^-production [22], but our observations specifically implicate NO· in the creation of excess AP sites requiring Xth or Nfo for repair. AP endonuclease activity is dispensable for *Salmonella* during infection of L-NIL treated mice (Figure 2), although L-NIL inhibits only NO· but not O_2_^−^ production.

NO· is able to mediate the deamination of DNA bases that contain primary amines: cytosine, guanine and adenine [8]. However, inactivation of the *alkA-* and/or *tag-*encoded glycosylases does not affect the NO·-sensitivity of *xth nfo* mutants, implying that glycosylase-mediated removal of deaminated dG (dX) or dA (dHX) does not contribute significantly to NO· generation of AP sites (Figure 1C). This may be because dX and dHX can also be removed from NO·-damaged DNA by Endonuclease V (*nfi*), a redundant repair enzyme that does not require additional AP endonuclease processing [34]. In contrast, no backup pathway exists for the removal of chromosomal dU produced by cytosine deamination, so the repair of NO·-deaminated dC relies exclusively on the Ung glycosylase and subsequent processing by Xth or Nfo. This accounts for the particular importance of dU in the sensitivity of *xth nfo* mutant *S.* Typhimurium to NO·.

The deamination of dC by NO· is not the only potential source of increased genomic dU requiring BER. The *dut* gene encodes a dUTPase enzyme responsible for minimizing the steady state concentration of dUTP in the free nucleotide pool. Mutations in *dut* result in increased DNA misincorporation of dUTP. The role of AP endonucleases in removing dU from misincorporation is demonstrated by the synthetic lethality of *dut*-1 and *xth* mutations [35]. In a *dut*-1 background, the Ung glycosylase eliminates misincorporated dU in DNA but generates AP sites that are repaired by Xth. Accordingly, the conditional lethality of *dut*-1 and *xth* mutations can be ameliorated by the inactivation of *ung* [36]. Interestingly, many other DNA repair genes are also synthetically lethal when combined with a *dut*-1 mutation (including *xth*, *polA*, *lig*, *recA*, *recBC*, and *ruvABC*) [36]. Mutations in many of these same genes increase sensitivity to NO· *in vitro* and attenuate bacterial virulence [21],[37]. Thus, dUTP misincorporation in a *dut*-1 mutant simulates the increased dU resulting from NO·-mediated dC deamination. Both conditions result in an excess of AP sites following Ung-mediated removal of chromosomal dU, and thus both conditions require intact BER and recombinational repair for viability.

NO·-dependent DNA damage is not limited to base deamination. The products of activated macrophages can oxidize chromosomal dG bases at the C8 position, primarily generating 8-oxoG (8-oxoguanine) and Fapy (formamidopyrimidines) [38]. NO·, when combined with superoxide to form the potent oxidant peroxynitrite, can potentiate the formation of 8-oxo-G and Fapy [24],[37]. 8-oxoG and Fapy are the predominant substrates for the Fpg glycosylase, as are some forms of oxidized pyrimidines (e.g., uracil glycol, 5-hydroxycytosine and 5-hydroxyuracil) unassociated with NO·-exposure. The partial restoration of NO·-resistance and virulence in *xth nfo* mutant *S.* Typhimurium following the inactivation of *fpg* is consistent with the generation of AP sites by Fpg-mediated excision of NO·-damaged dG. This implies that some Fpg activity is present in NO·-exposed *S.* Typhimurium during infection, despite the NO·-labile zinc-finger domain of Fpg [28]. Furthermore, these data suggest that sufficient concentrations of peroxynitrite are generated to result in dG oxidation *in vivo*. 8-oxoG, together with deaminated DNA bases, accounts for a major portion of the glycosylase-mediated AP sites generated by host NO· exposure.

While the NO·-sensitivity of *xth nfo* can be used as a probe to identify the types of base damage resulting from nitrosative stress, an intact BER pathway protects *S.* Typhimurium from the cytotoxic effects of host NO·. Nevertheless, high concentrations of nitrogen oxides are mutagenic for wild type *S.* Typhimurium. Exposure of *S.* Typhimurium in culture to NO· released from 1 mM SperNO increases the measurable mutation rate by nearly 14-fold (Table 1). A significant proportion of NO·-induced mutation is limited by Ung-mediated removal of dU. Of the two sources of chromosomal dU, dUTP incorporation is far more abundant (10^5^-fold) than spontaneous dC deamination [39], but is not mutagenic because dUTP only pairs with dA. The tendency of *dut*-1 *ung* mutants to accumulate significant chromosomal dU levels (nearly 1 dU residue per 125 nucleotides) without severe physiological consequences demonstrates that chromosomal dU is not inherently detrimental to the cell [40],[41]. However, the mutagenic potential of deaminated dC provides a strong selective pressure for the presence of Ung activity. Indeed, *ung* mutants possess inherent increased baseline mutability resulting from spontaneous dC deamination that is exacerbated by the addition of NO· (Table 1) [42]. Furthermore, *S.* Typhimurium *ung* mutants exhibit a 12-fold increase in 5-FC resistance rates compared with wild type cells during infection (Figure 3). This *in vivo* hypermutability is the direct result of NO·-mediated dC deamination, as the inhibition of murine NO·-production eliminates excess mutability associated with the *ung* mutation (Figure 3). Nevertheless, it is also noteworthy that wild type *Salmonella* does not exhibit NO·-dependent hypermutability during infection, indicating that Ung and BER are sufficient to remove excess deaminated dC arising from host NO·-exposure.

**Figure 4:**
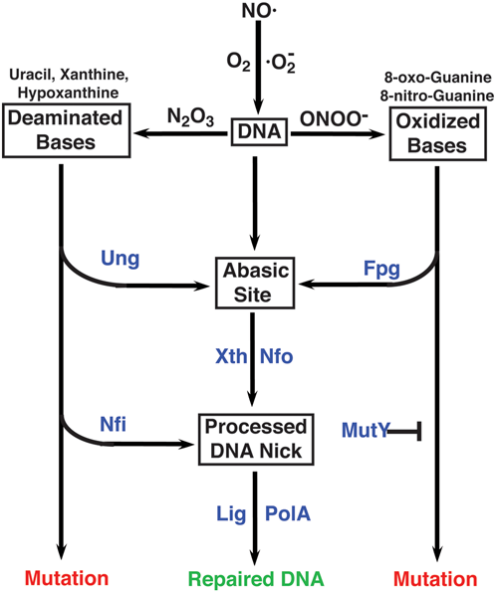
Conceptual representation of mutation avoidance by BER during nitrosative stress. NO· can be oxidized to nitrous anhydride (N_2_O_3_) and/or peroxynitrite (ONOO^−^) depending in the presence or absence of superoxide. Nitrous anhydride and peroxynitrite mediate DNA base deamination and oxidation, respectively. The Ung repair pathway removes dU from DNA via an AP site, thereby avoiding the MUTATION pathway. Similarly, Fpg removes oxidized dG, limiting peroxynitrite-mediated hypermutation, but increased mutability in cells lacking Fpg can be ameliorated by MutY. NO·-induced mutations can also be avoided by pathways that do not produce AP site intermediates (e.g., Nfi).

In addition to its cytotoxic effects, 8-oxoG can also be mutagenic resulting from its propensity to form 8-oxo-dG:dA pairs. However, the contribution of oxidized dG to overall NO·-induced hypermutability in broth cultures *in vitro* appears to be less than that of deaminated dC. Specifically, NO·-exposed *ung* cells in broth culture exhibit elevated mutability compared with wild type bacteria, but NO·-treated *fpg* mutants do not (Table 1). This can be rationalized by the existence of other DNA glycosylases that protect against 8-oxoG-associated mutations, e.g., MutY and Nei [43]. MutY specifically removes dA paired to 8-oxoG, and Nei can also remove chromosomal 8-oxoG, albeit with lower efficiency. Furthermore, the low production of peroxynitrite in aerated NO·-treated broth cultures would be anticipated to reduce the frequency of 8-oxoG-derived mutations. Indeed, *fpg* mutants exhibit significantly greater mutability than wild type cells upon direct exposure to the peroxynitrite generated following the addition of SIN-1 to highly aerated cultures. Moreover, during infection when *Salmonella* is exposed to high levels of both NO· and reactive oxygen species, the role of Fpg in avoiding NO·-mediated hypermutation is readily apparent (Figure 3). *S.* Typhimurium *fpg* mutants exhibit a 21-fold increase in 5-FC resistance rates during infection compared with wild type cells. Whereas host-derived NO· is solely responsible for the *in vivo* hypermutability observed in *ung* mutant bacteria, iNOS inhibition with L-NIL in mice infected with *fpg* mutant *S.* Typhimurium fails to eliminate all excess bacterial mutability (Figure 3). Damaged DNA base substrates for Fpg can be created by reactive oxygen species independently from peroxynitrite, accounting for the residual hypermutability of *fpg* mutant bacteria in L-NIL treated mice. These observations suggest that *Salmonella* growing in an immunocompetent host encounter peroxynitrite, but the bacterium is capable of minimizing the mutagenic effects of this oxidant through the actions of Fpg and the BER pathway (Figure 4).

Collectively, this work demonstrates that the *Salmonella* chromosome incurs high levels of base deamination and guanine oxidation during infection as a result of host NO· production, but BER limits the mutagenic effects associated with these DNA modifications (Figure 4). The function of BER is not to prevent bacterial killing by NO·, because cytosine deamination and guanine oxidation are not lethal *per se*, but rather to preserve genomic fidelity. While it continues to be debated whether enhanced mutation rates can be adaptive under stress conditions [44], our observations indicate that BER allows *S.* Typhimurium to maintain a low mutation rate despite the stressful intracellular environment. It has been suggested that host DNA damage resulting from inflammation may lead to permanent tissue damage, chromosomal lesions and cancer [37],[45]. Our studies suggest that an important human pathogen is able to use a highly conserved DNA repair pathway to limit the genotoxic effects of NO· generated by the host inflammatory response.

## Materials and Methods

### Strains and culture conditions

Bacteria used in this study are derivative of *S.* Typhimurium 14028s and are listed in Table 2. Strains were routinely grown in Luria-Bertani (LB) broth and the following antibiotics were added when appropriate: penicillin G (250 μg·ml^−1^) kanamycin (50 μg·ml^−1^), and chloramphenicol (40 μg·ml^−1^). When indicated, culture medium was supplemented with 1 mM concentrations of the NO· donor Spermine/NONOate (SperNO) (Cal Biochem) or 4 mM concentrations of the peroxynitrite-generator SIN-1 (A.G. Scientific, Inc.). Mutant *S.* Typhimurium strains were constructed using the λ−red method (Table 2) [46]. Each mutation was confirmed by PCR analysis using gene-flanking primers (Table 2), then transduced via P22 phage back into wild type 14028s.

**Table 2:** Bacterial strains and DNA primers used in this study.

Bacterial strains used		
Strain	Source	Genotype
*S.* Typhimurium		
14028s	ATCC	Wild Type
PB403	This Study	*fpg*::Cm^R^
JS1018	This Study	*ung*::Cm^R^
PB412	This Study	Δ*xth*Δ*nfo*
KS55	This Study	Δ*xth*Δ*nfo alkA*::Km^R^
PB523	This Study	Δ*xth*Δ*nfo nei*::Km^R^
PB415	This Study	Δ*xth*Δ*nfo nth*::Km^R^
PB417	This Study	Δ*xth*Δ*nfo fpg*::Cm^R^
PB512	This Study	Δ*xth*Δ*nfo ung*::Cm^R^

Primers used	
λ-red primers (5′-3′)	
AlkA for:	atgtttaccctgagctggcagccgccttacgagtgtaggctggagctgcttc
AlkA rev:	gaataccagctatttcgctatccattgacgcatatgaatatcctccttag
Fpg1A:	cgaagttgaaaccagccgccggggtattgaaccccatttagttggcgctactatcctgcagtctaggctggagctgcttc
Fpg1B:	caatagaacgtcgcgcgctgtgcgtgtttcgttgcgactatcggcgtaccgcatacccgacatatgaatatcctccttag
Nei1A:	ctgaaggcccggaaattcgtcgtgcggcggataatctggaggcggcaatcaaaggcaaacgtgtaggctggagctgcttc
Nei1B:	ctggcaatgcgggcaccagtaaaacgggcgtgaggacagtgttgtcttttcaataatccgcatatgaatatcctccttag
Nfo1A:	catcggagcgcacgtcagcgctgctggcggtctggctaacgcccctgcccgcgcggctgagtgtaggctggagctgcttc
Nfo1B:	catcggagcgcacgtcagcgctgctggcggtctggctaacgcccctgcccgcgcggctgagtgtaggctggagctgcttc
Nth1A:	caaagcaaaaaggctggaaattctgacgcgcctgcgcgacaacaatccgcaccccacaacgtgtaggctggagctgcttc
Nth1B:	ctttttctttgtattcgcaaagatcttcaatgaggcaggagccgcagcggggttttcgggcatatgaatatcctccttag
Ung1A:	cgcttcacactgttgctggcgagcgtcagtcgggtatcacggtctacccgccgcaaaaaggtgtaggctggagctgcttc
Ung1B:	cgtccagtcgatagttttctcgccgtgttgttccagccattggttcgttagcgcaaaatgcatatgaatatcctccttag
XthA1A:	gtctcttttaatatcaacggcctgcgcgcccgtccccatcagcttgaagcgattgtcgaagtgtaggctggagctgcttc
XthA1B:	cgcccagaccggcgcatggtcggagggtttttccatactgcgaatgtcgtagtctatacccatatgaatatcctccttag
Diagnostic Primers (5′-3′)	
Ung2A:	gcttcacactgttgctggcgagcgtcagtgtcgggtatcacggtctacccgccgcaaaaagaaagccctggagcac
Ung2B:	aaatggttacagccaaaaaatccgcggtcggcagagagtggcgagggatgc
Fpg2A:	cggagacgttatgcctgaatt
Fpg2B:	cctggcattttacttctgaca
Nth2A:	caaagcaaaaaggctggaaat
Nth2B:	caaatatcgactttttctttg
Nei2A:	gcctgaaggcccggaaattcgtcg
Nei2B:	ctggcaatgcgggcaccagta
Xth2A:	gcaaagtggcagtgactatga
Xth2B:	cagaaagcggaatcaaacacg
Nfo2A:	gggtttaacaggagtctttta
Nfo2B:	gcctgaagaacattgagcgtc

### NO· sensitivity determination

Growth kinetics following NO·-exposure was determined by measuring optical density at 600 nm at 37°C using a Bioscreen C incubator/reader (Growthcurves USA). Overnight culture were diluted to 1:100 in LB medium and grown to early log phase (OD_600_ ∼0.2) before the addition of 1 mM SperNO. For cell viability determination, bacterial samples were taken hourly and serially diluted and plated onto LB agar following NO· exposure. Colony forming units (cfu) were scored after incubation at 37°C for 18 hrs.

### Virulence determination

Six to eight week old CH3/HeN mice (Charles River Laboratories) were inoculated intraperitoneally with 1×10^3^ cfu of wild type and isogenic mutants *S*. Typhimurium strains. Survival was monitored in twelve mice were used for each tested condition. The mice were checked twice daily for 28 days and moribund mice sacrificed per IACUC protocol. Competitive defects in mutant bacteria were also assessed in murine liver and spleen tissue from 5 mice each on days 3, 5, 7 and 9 post-infection. Tissue was homogenized and plated on appropriate antibiotics. Competitive indices were determined as the ratio (mutant:WT)_OUT_ to (mutant:WT)_IN_. To selectively inhibit NO· production by NOS2, L-NIL [L-N^6^-(1-iminoethyl)-lysine] was administered via drinking water at 500 μg·ml^−1^. L-NIL has been shown to have ∼30 fold selectivity for the inhibition of NOS2 over other NOS isoforms [30].

### Mutability determination

*In vitro* mutation frequency of wild type and mutant *S*. Typhimurium was measured as the rate of spontaneous rifampin-resistance (resulting from mutations in *rpoB*). Overnight cultures of *Salmonella* were diluted 1:100 in 5 ml of fresh LB exposed either to 1 mM SperNO or 4 mM SIN-1. SperNO treated strains were grown in 18 mm test tubes, while SIN-1 treated strains were grown in 250 ml Erlenmeyer flasks to maximize aeration. All bacterial cultures were grown overnight at 37°C and plated onto LB agar with or without 100 μg·ml^−1^ rifampin (Fisher Scientific). *In vivo* mutability was determined by infecting C3H/HeN mice with 1×10^3^ cfu of wild type or isogenic mutant *S*. Typhimurium. When appropriate, mice were treated with the NOS2 inhibitor L-NIL administered via drinking water at 500 μg·ml^−1^. Mice were monitored for seven days and their livers were harvested, homogenized in 1 ml of PBS and viable cfu/g liver determined. Appropriate dilutions were also plated onto M9 agar containing 60 μg·ml^−1^ 5-fluorocytosine (5FC) (Sigma-Aldrich). Homogenates were grown for 48 hrs at 37°C, then replica plated onto fresh 5FC plates for further incubation at 37°C overnight. Mutation frequency was calculated from number of cfu on 5FC plates divided by the total number of viable cfu per g liver.
